# *Potato spindle tuber viroid* infection triggers *degradation of chloride channel protein CLC-b-like* and *Ribosomal protein S3a-like* mRNAs in tomato plants

**DOI:** 10.1038/s41598-017-08823-z

**Published:** 2017-08-21

**Authors:** Charith Raj Adkar-Purushothama, Pavithran Sridharan Iyer, Jean-Pierre Perreault

**Affiliations:** 1 Département de Biochimie, RNA Group/Groupe ARN, Faculté de médecine des sciences de la santé, Pavillon de Recherche Appliquée au Cancer, Université de Sherbrooke, 3201 rue Jean-Mignault, Sherbrooke, Québec, J1E 4K8 Canada; 2 Département de Physique, Université de Sherbrooke, 2500 Boulevard de l’Université Sherbrooke, Québec, J1K 2R1 Canada

## Abstract

It is well established that viroid derived small RNA (vd-sRNA) induces RNA silencing of endogenous mRNA. However, it remains not clear how exactly viroid infections can lead to severe symptom induction given the fact that fewer vd-sRNAs binding the specific target mRNAs were recovered from the infected plants. To answer this question, the two least expressed (+) and (−) strand vd-sRNAs of potato spindle tuber viroid (PSTVd) binding to both the 3′ UTR and the coding region of tomato mRNAs were analyzed by infecting tomato plants with two variants of PSTVd. As products of these putative target mRNAs are involved in plant phenotype, the effect of this viroid on these genes were analyzed by infecting tomato plants with two variants of PSTVd. The direct interaction between the vd-sRNAs and putative mRNAs was validated by artificial microRNA experiments in a transient expression system and by RNA ligase-mediated rapid amplification of cDNA ends. Parallel analysis of RNA ends of viroid infected plants revealed the widespread cleavage of the target mRNAs in locations other than the vd-sRNA binding site during the viroid infection implying the viroid-infection induced vd-sRNA independent degradation of endogenous mRNAs during viroid infection.

## Introduction

Viroids are non-coding, highly structured, circular, single-stranded RNA (ssRNA) molecules of 246–401 nucleotides (nts) in length^1^. To date, 32 species of viroid have been described, and broadly classified into two families: the *Avsunviroidae* and the *Pospiviroidae* ^2^. The members of the family *Avsunviroidae*, whose type species is the *Avocado sunblotch viroid* (ASBVd), replicate in chloroplasts through a rolling-circle mechanism and exhibit self-cleavage activity. In contrast, members of the family *Pospiviroidae*, whose type species is the *Potato spindle tuber viroid* (PSTVd), replicate in the nucleus and contain five structural/functional domains: the Terminal Left (TL), Pathogenicity (P), Central (C), Variable (V) and Terminal Right (TR) domains^3, 4^.

RNA interference (RNAi) regulates gene expression both transcriptionally and post-transcriptionally^5^. Either the double-stranded RNA (dsRNA) or the self-complementary RNA triggers RNA silencing through the activity of the DICER*-like* (DCL) *RNase* III-type ribonucleases, and results in the production of small interfering RNAs (siRNA). These small RNAs (sRNA) comprise the sequence-specific effectors that regulate gene expression^6^. Due to their high internal base pairing and RNA-RNA mode of replication, viroids are the elicitors of the host defense system via RNA silencing. As a result, viroid derived sRNA (vd-sRNA), which are 21- to 24-nts long, are often recovered from viroid infected host plants^7–9^. Previously, it has been shown that the (−) sRNAs derived from *Peach latent mosaic viroid* (PLMVd) can target the coding region of the *heat shock protein 90* (cHS90) in peach plants (*Prunus persica*), and the (+) sRNA derived from the virulence modulating region (VMR) of PSTVd can down-regulate soluble inorganic *pyrophosphatase* gene in *Nicotiana species* and *callose synthase* gene in tomato^10–13^. However, to date there is no explanation for the higher level of down-regulation of endogenous mRNAs in the presence of fewer amounts of recovered target binding vd-sRNAs.

PSTVd is a good model for studying viroid-host interactions, as it is a widely-studied viroid^12, 14^. For example, previously it has been used to demonstrate the viroid’s (i) structure in solution by high-throughput selective 2′-hydroxyl acylation analyzed by primer extension (SHAPE)^3^, (ii) cell-to-cell trafficking through plasmodesmata and its systemic movement through phloem^15^, (iii) role of vd-sRNA in different host species such as tomato and *Nicotiana* species^11–13^, and, (iv) to develop viroid resistant plants^16, 17^. It has also been used to monitor tomato gene regulation by microarray analysis and its effect on tomato miRNA expression^9, 18^. In addition, the availability of the tomato genome allows for prediction of sRNA target genes^19^. In this study, the least expressed (+) and (−) PSTVd-sRNA were selected as a starting point in order to analyze the fate of their target mRNA during PSTVd infection in tomato plants using both computational and molecular biology approaches.

## Results

### Selection of the vd-sRNAs to be used for target prediction

**Figure 1:**
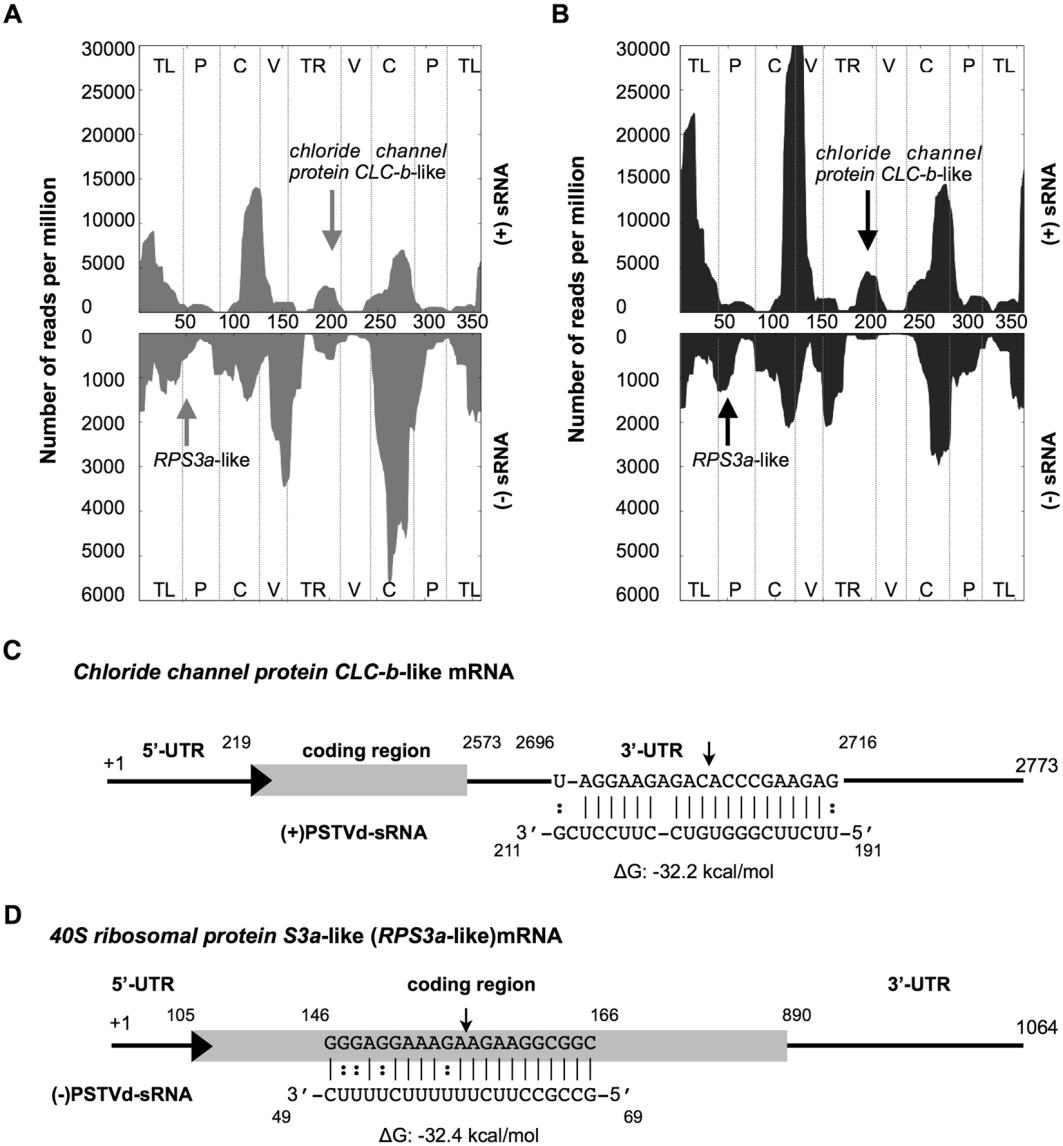
Profiling of the vd-sRNAs recovered from PSTVd infected tomato plants and the predicted vd-sRNA:target duplexes. Sequence profiles of the (+) and the (−) PSTVd-sRNA populations recovered from the leaf tissues of infected tomato plants. Panels (A) and (B) represent the profiles of the (+) and the (−) PSTVd-I derived sRNAs, while panels (C) and (D) represent the profiles of the (+) and the (−) PSTVd-RG1 derived sRNAs. The vertical arrows denote the two vd-sRNA populations of particular interest, namely those capable of targeting potential host mRNAs: a (+) vd-sRNA located at positions 119 to 211 and a (−) vd-sRNA located at positions 69 to 49. Please note that different scales are used so as to compensate for the lower numbers of (−) vd-sRNA sequences recovered for both of the PSTVd variant infected plants. The predicted interactions between the PSTVd-sRNAs derived from the (**C**) (+) strand with the 3′ UTR of the *chloride channel protein CLC-b-like* mRNA and (**D**) the (−) strand with the coding region of the *40S ribosomal protein S3a-like* mRNA are shown. The arrows indicate the predicted RISC mediated cleavage sites. The sequences are shown in the complementary polarity. The PairFold online tool was used to predict the minimum free energy (ΔG) secondary structures of the pairs of RNA sequences.

In order to elucidate the most susceptible regions of both the PSTVd (+) and (−) strands for RNA silencing, the vd-sRNA profiles obtained at 21 days post inoculation (dpi) from tomato plants (*Solanum lycopersicum* cv. Rutgers) infected with PSTVd variants (PSTVd-intermediate [PSTVd-I]; PSTVd-RG1) were analyzed so as to identify the regions producing the sRNA of 21 to 24-nt in length (Table 1). Profiling of vd-sRNA revealed that certain regions of the PSTVd genome produced more sRNAs than others, that is to say, they are vd-sRNA producing hotspots (produces more than 10% of sRNA) while less vd-sRNA producing regions on viroids are called as non-hotspots (produces less than 10% of sRNA; Fig. 1A); for details see refs 16, 20 and 21. In order to investigate the possible vd-sRNA derived from the PSTVd variants binding to 3′ UTR targets in tomato transcriptome datasets, the 21-nt long vd-sRNA were then analyzed using the psRNATarget web-based tool^22^. For each predicted vd-sRNA:target duplex, the value of Gibbs free energy, represented as ∆G, was calculated using the PairFold web-based tool^23^. Interestingly, sRNA derived from the lower TR of the (+) strand of PSTVd was predicted to target the 3′ UTR of the *chloride channel protein CLC-b-like* mRNA (GenBank Acc. No. XM_004233252; Fig. 1B). Chloride channels are involved in physiological functions in almost every cell ranging from prokaryotes to eukaryotes^24, 25^. Analysis of the sRNA data revealed that a total of 225 (0.3%) and 196 (0.26%) vd-sRNAs targeting the *chloride channel protein CLC-b-like* mRNA in PSTVd-I and PSTVd-RG1 infected plants were obtained, respectively. None of the sRNA derived from healthy plants targeted *chloride channel protein CLC-b-like* mRNA (Fig. 1C). Similar investigation for the involvement of (−) vd-sRNA in viroid pathogenicity, the P domain region was selected because it is known to be involved in viroid induced disease symptoms^26^. The analysis of the P domain of the (−) strand of PSTVd as described above was predicted to bind the coding region of the *40S ribosomal protein S3a-like* mRNA (*RPS3a-like*; GenBank Acc. No. XM_004241413; Fig. 1D). *RPS3a-like* is a structural constituent of the ribosome; hence, it is directly involved in translation (http://www.uniprot.org/uniprot/P49397). This protein has also been shown to interact with the DNA damage-inducible transcript 3^27^. Analysis of sRNA data obtained for tomato plants infected with PSTVd variants revealed only 4 (0.005%) and 8 (0.01%) vd-sRNAs, targeting the *RPS3a-like* mRNA from the (−) strands of PSTVd-I and PSTVd-RG1 in infected plants, respectively, but none from healthy plants.

**Table 1:** Potato spindle tuber viroid (PSTVd)-sRNA reads found in the tomato cv. Rutgers leaf tissue infected with PSTVd-I and PSTVd-RG1.

Sample	Total sRNA	PSTVd-sRNA	% PSTVd-sRNA	(+) PSTVd-sRNA	(−) PSTVd sRNA	(+)/(−) PSTVd-sRNA ratio
**Control**	567,803	31	N.A.*	26	5	N.A.*
**PSTVd-I**	1,201,086	74,748	6.22	51226	23522	2.2
**PSTVd-RG1**	730,499	74,916	10.25	64644	10272	6.3

*Not applicable.

### Role of predicted target genes on host phenotype

**Figure 2:**
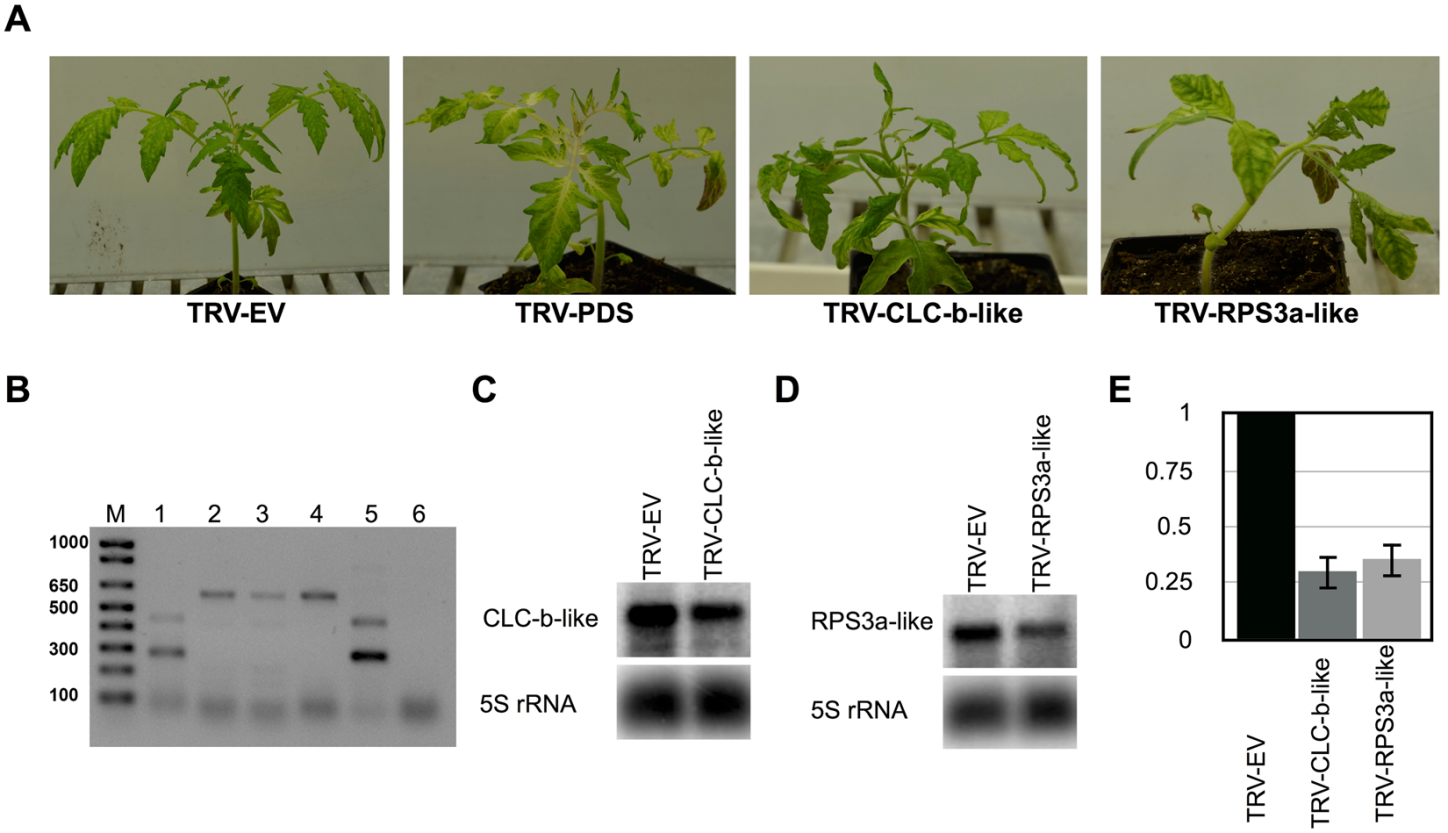
Effect of *chloride channel protein CLC-b-like* mRNA and the *40S ribosomal protein S3a-like* mRNA on the plant’s morphology. (**A**) The tomato plants were subjected to a knock-down assay by a VIGS technique using the TRV1 vector in combination with either the TRV2 empty vector, or with its derivatives. After 18-days post infiltration plants exhibited phenotypic alterations similar to viroid infection. TRV-EV, TRV empty vector inoculated plants; TRV-PDS, plants inoculated with pTRV2:PDS; TRV-CLC-b-like, the plant infiltrated with pTRV2:CLC-b-like; and, TRV-RPS3a, the plant infiltrated with pTRV2:RPS3a. (**B**) The total nucleic acids extracted from these plants were PCR analyzed for the systemic presence of TRV2 derivatives. Lane M, 1 kb plus molecular marker, Lane 1, TRV-EV; Lane 2, pTRV2:PDS; Lane 3, TRV-CLC-b-like; Lane 4, TRV-RPS3a; Lane 5, PCR positive control for the TRV-EV vector; and, lane 6, PCR negative control. Total RNA extracted samples from the agroinfitrated plants were analyzed by gel blot assay for the knock-down/suppression of (**C**) *Chloride channel CLC-b-*like and, (**D**) *RPS3a*-like mRNAs using gene specific radiolabeled probes. The *5 S* rRNA was used as a loading control. Full size gel blots are presented in Fig. S1. (**E**) The gel blot signals from (**C**) and (**D**) were quantified and expressed as a ratio of the target mRNA to the 5S rRNA signals. For each set of experiments, the ratio of target mRNA to 5S rRNA obtained with TRV-EV (control) was set at a value of 1. The additional bars indicate the relative target mRNA/5S rRNA ratio for each TRV-derivate vector (as indicated) expressed. Each experiment was performed at least three times. Error bars indicate SD.

In order to verify the role of *chloride channel protein CLC-b-like* and the *RPS3a*-*like* mRNA products on the host’s morphology, the virus induced gene silencing (VIGS) assay was performed on tomato plant. Approximately 400-nt long *chloride channel protein CLC-b*-*like* and *RPS3a*-*like* genes were expressed in the pTRV2 vector under the control of the 35S promoter. The resulting binary vectors were named pTRV2:CLC-b-like (targets the *chloride channel protein CLC-b*-*like* mRNA) and pTRV2:RPS3a (targets the *RPS3a*-*like* mRNA). pTRV1 and pTRV2, or their derivatives, were then transformed into an *Agrobacterium tumefaciens*, and were then used for agroinfiltration into tomato seedlings as previously described^28^. pTRV2:PDS, which is capable of targeting Phytoene desaturase mRNA (*PDS)*, was used as a positive control, while a negative control was maintained by agro-infiltrating the plants with empty pTRV2 vector. At approximately 18-dpi, all the virus induced gene silenced (VIGSed) plants exhibited phenotypic alterations when they were compared to control plants (Fig. 2A). The pTRV2:CLC-b-like VIGsed plants showed mild stunting and leaf curling, while the plants agro-infiltrated with pTRV2:RPS3a were severely stunted and demonstrated a reduction in overall development. The presence of the agroinfiltrated pTRV2 derivatives in plants was confirmed by PCR assay (Fig. 2B). The down-regulation of the expression of each targeted gene was further confirmed by RNA gel blot assay (Fig. 2C,D and Fig. S1). The data presented here demonstrate that both the *chloride channel protein CLC-b*-*like* and the *RPS3a*-*like* mRNAs products are crucial for the overall development of the plant.

### PSTVd infection down-regulates the predicted target genes

**Figure 3:**
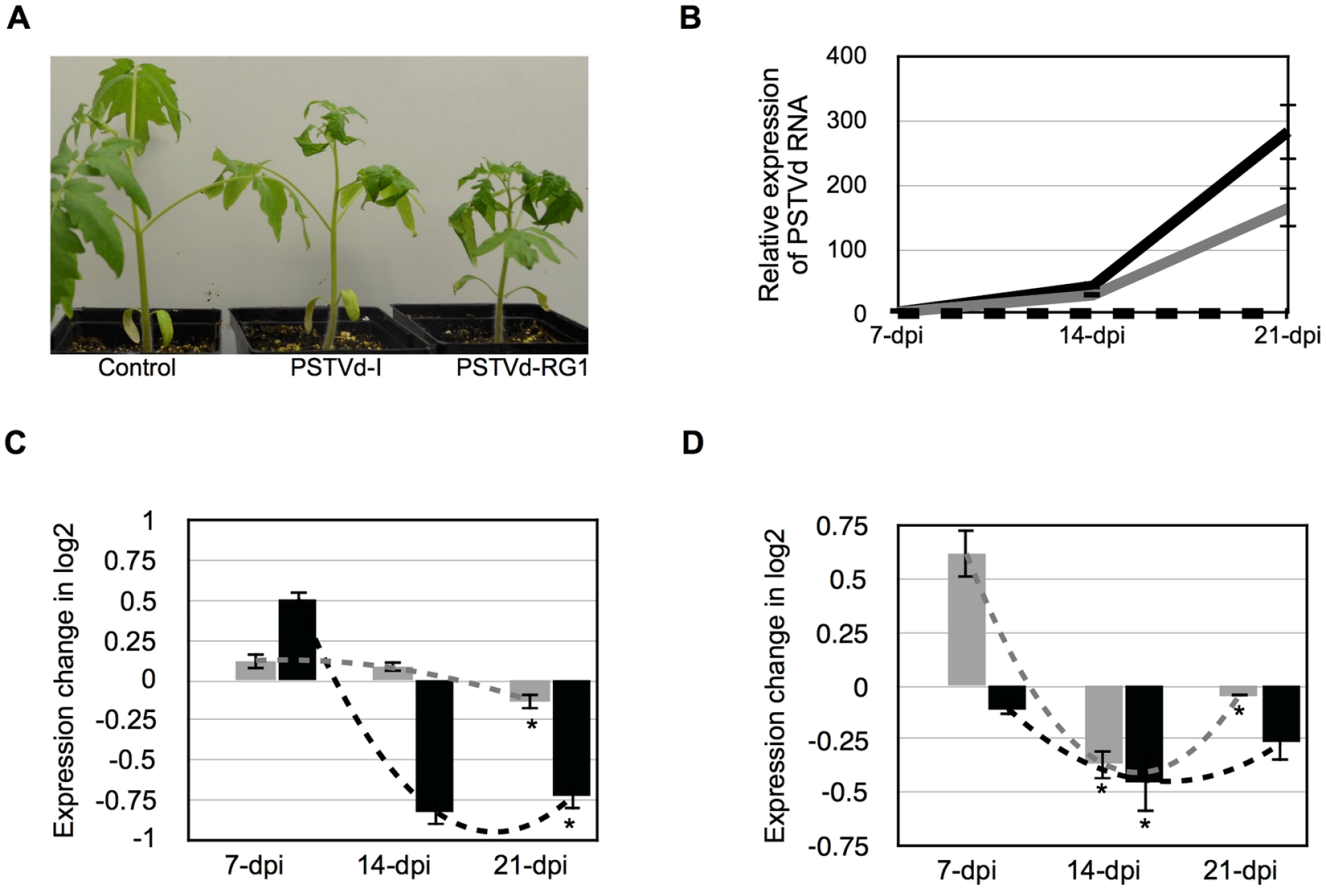
Effect of the PSTVd variants on the predicted target mRNAs. (**A**) Both the PSTVd-I and PSTVd-RG1 variants were inoculated into tomato plants. At 14-dpi, the plants inoculated with both of the PSTVd variants showed disease symptoms when compared to mock inoculated plants. (**B**) Total RNA extracted from tomato plants at 7, 14 and 21-dpi were used to monitor the PSTVd titer. In the graph, the dotted black line (on the X-axis) represents mock inoculated plants, while the grey and black solid lines indicate the PSTVd-I and PSTVd-RG1 inoculated plants, respectively. The effects of the PSTVd variants on the levels of the (**C**) *chloride channel protein CLC-b-like* and (**D**) the *RPS3a-like* mRNAs were evaluated at different time intervals. The expression change is presented on a log_2_ scale. Each experiment was performed at least three times with true biological replicates. The changes in the expression levels of the mRNAs between the time points are shown with dotted lines. The error bars indicate SD. The asterisks indicate statistically significant for paired *t-*test (*P* < 0.05).

To verify the effect of PSTVd infection on both the *chloride channel protein CLC-b-like* and *RPS3a-like* mRNAs, bioassays were performed in tomato plants at different time intervals using two different PSTVd variants, namely PSTVd-I and PSTVd-RG1. Upon infection, both variants induced severe disease symptoms in tomato plants. The tomato plants inoculated with either PSTVd-I or PSTVd-RG1 exhibited initial disease symptoms such as leaf curling and stunted growth at 10 to 12-dpi (Fig. 3A). In order to monitor the accumulation of both viroids, leaf samples were collected at 7, 14, and 21-dpi and used to extract total RNAs. The viroid levels at the different time intervals were then evaluated by reverse transcription-quantitative polymerase chain reaction (RT-qPCR) on the total RNA as described previously^12^. The RNA levels of both PSTVd variants increased rapidly, but PSTVd-RG1 tended to accumulate much faster and reached almost twice the titer of PSTVd-I at 21-dpi (Fig. 3B).

In order to determine the effect of the different PSTVd variants on the expression levels of the *chloride channel protein CLC-b-like* and the *RPS3a-like* mRNAs, RT-qPCR analysis was performed on the 7, 14 and 21-dpi RNA samples (which had previously been used to quantify viroid accumulation) using gene specific primers. As presented in Fig. 3C, PSTVd-RG1 infection negatively affected the *chloride channel protein CLC-b-like* mRNA level at 14-dpi, PSTVd-I down-regulated it at 21-dpi. On the other hand, both PSTVd variants down-regulated the *RPS3a-like* mRNA level at 14 and 21-dpi (Fig. 3D). More specifically, PSTVd-RG1 infected plants showed a higher level of repression than did those infected with PSTVd-I. Taken together, this data suggests that PSTVd infection has the potential to down-regulate both the *chloride channel protein CLC-b-like* and the *RPS3a-like* mRNA levels.

### PSTVd derived sRNAs favor the RNAi-mediated cleavage of the predicted target genes

**Figure 4:**
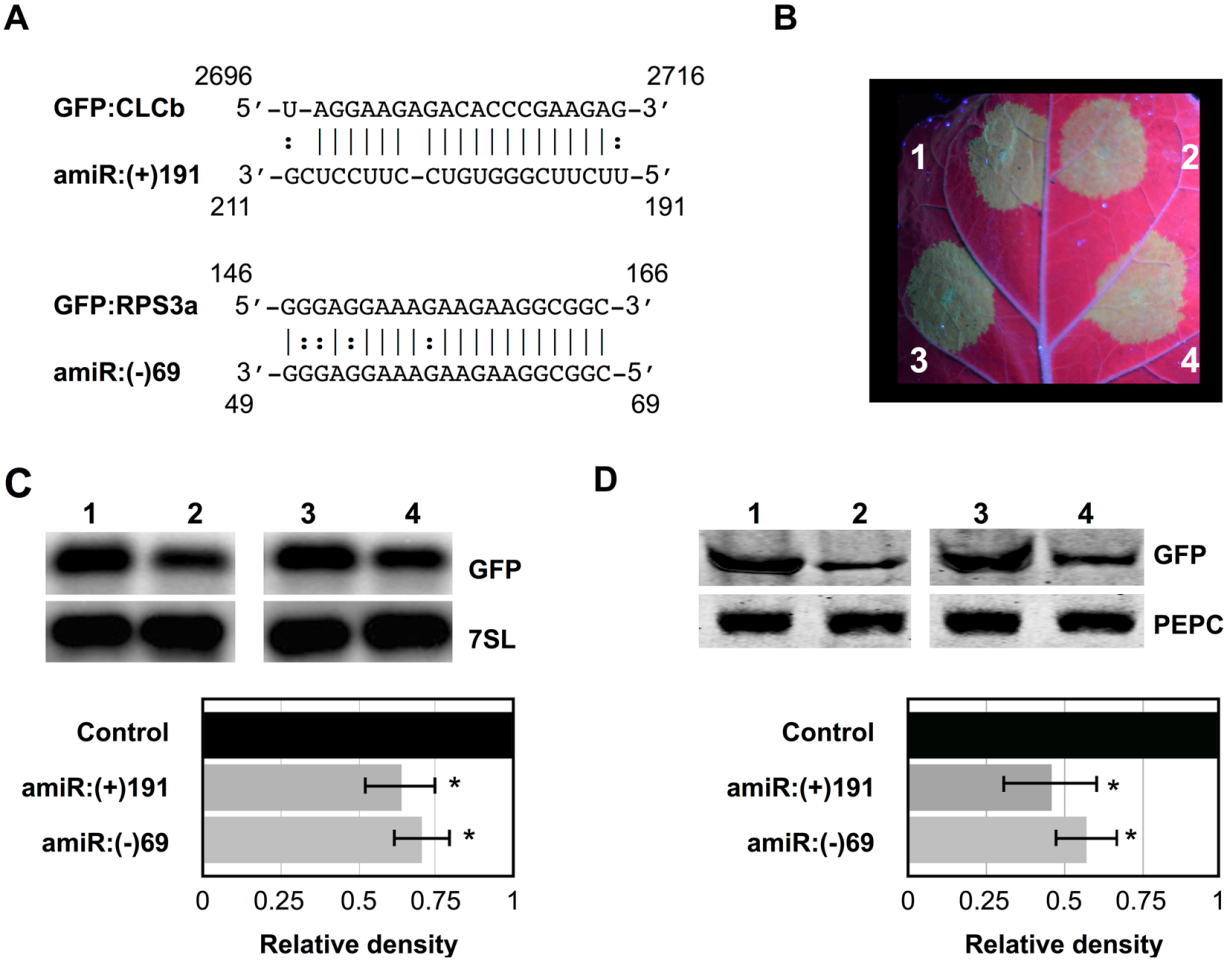
Validation of the predicted vd-sRNA:target mRNA complex formation by an artificial microRNA. (**A**) Duplexes predicted to be formed by complexes of amiRNAs and GFP reporter constructs containing the *Chloride Channel CLC-b-like* and the *RPS3a-like* mRNA target sequences. (**B**) *N. benthamiana* leaves were agro-infiltrated with (1) empty pBIN61 vector (EV) plus GFP:CLCb; (2) amiR:(+)191 plus GFP:CLCb; (3) EV plus GFP:RPS3a; and, (4) amiR:(−)69 plus GFP:RPS3a. At 3-dpi, the leaves were photographed under UV illumination. *N. benthamiana* leaves were agroinfiltrated in the same combinations as in (**B**). (**C**) At 3-dpi, total RNA extracts were subjected to RNA gel blot analyses with either GFP (top panel) or *7SL* (lower panel) radiolabeled probes. Full size gel blots are presented in Fig. S2. The signals in (**C**) were quantified and expressed as a ratio of the GFP to the *7SL* signals. For each set of experiments, the ratio of GFP to *7SL* obtained with EV plus GFP:XX (control) was set at a value of 1. The additional bars indicate the relative GFP/*7SL* ratio for each amiRNA (as indicated) expressed with its respective GFP:XX. (**D**) At 3 dpi, total protein extracts were subjected to immunoblotting with anti- GFP (top panel) and anti-PEPC (lower panel) antibodies. Full size immunoblots are presented in Fig. S3. The immunoblot signals from (**D**) were quantified and expressed as a ratio of the GFP to the PEPC signals. For each set of experiments, the ratio of GFP to PEPC obtained with EV plus GFP:C11-vdXX (control) was set at a value of 1. The additional bars indicate the relative GFP/PEPC ratio for each amiRNA (as indicated) expressed with its respective GFP target. Each experiment was performed at least three times. Error bars indicate SD. The asterisks indicate statistically significant for paired *t-*test (*P *<  0.05).

In order to demonstrate both the RNA/RNA interaction and the RNA induced silencing complex (RISC) mediated cleavage of both the *chloride channel protein CLC-b-like* and the RPS3a-like mRNAs, an artificial microRNA (amiRNA) system was developed for the transient expression of the vd-sRNA derived from positions 191 to 211 (5′-UUCUUCGGGUGUCCUUCCUCG-3′) of the (+) strand and positions 69 to 49 (5′-GCCGCCUUCUUUUUUCUUUUC-3′) of the (−) strand of PSTVd in *Nicotiana benthamiana* (*N*. *benthamiana*) leaves. All of the amiRNA constructs and the green fluorescent protein (GFP) tagged target constructs were created on pBIN61 vector as previously^12^. If the amiRNA produced vd-sRNA binds to the target sequence, the fluorescence level of the GFP will decrease as this binding blocks the translational process and/or affects the stability of the target mRNA^29^. The amiRNA construct which targets the *chloride channel protein CLC-b-like* mRNA was named amiR:(+)191; that targeting the *RPS3a-like* mRNA was named amiR:(−)69. The GFP constructs with the *chloride channel protein CLC-b-like* mRNA target sites were named GFP:CLCb, which is targeted by amiR:(+)191, and GFP:RPS3a, which is targeted by amiR:(−)69. The GFP constructs were co-agroinfiltrated in *N. benthamiana* leaves along with either their respective targeting amiRNAs or with empty vector. All of the vd-sRNA:target complexes predicted to be formed by the interaction between the amiRNAs and the putative mRNA target sequences are illustrated in Fig. 4A. At 3-dpi, the agro-infiltrated leaves were observed under UV illumination and GFP fluorescence was detected. All combinations of vd-sRNA:amiRNA plus GFP-target showed less GFP fluorescence than the same GFP:targets expressed with an empty vector, suggesting that the GFP-expressing mRNAs were targeted by the amiRNAs (Fig. 4B and Fig. S2). A RNA gel blot was performed on the total RNA extracted from the agroinfiltrated leaves using radiolabelled GFP riboprobe in order to verify the reduction in the amount of GFP-mRNA in the various vd-sRNA:amiRNA plus GFP:target combinations (Fig. 4C). The degree of GFP down-regulation by each vd-sRNA:amiRNA plus GFP:target combination was quantified and expressed as a relative density using the *7S signal recognition particle RNA* (*7SL*) mRNA level for normalization. In order to verify the amount of GFP produced in the various vd-sRNA:amiRNA plus GFP:target combinations, an immunoblot assay was performed using anti-GFP antibody (Fig. 4D and Fig. S3). The degree of GFP downregulation by each vd-sRNA: amiRNA plus GFP:target combination was quantified and expressed as relative density (Fig. 4D, lower panel) using the phosphoenolpyruvate carboxylase (PEPC) protein level for normalization. This experiment demonstrated the direct interaction between the vd-sRNAs derived from both strands of PSTVd with the predicted target mRNA sequences, as well as vd-sRNAs potential to induce the RISC mediated down-regulation of both the *chloride channel protein CLC-b-like* and the *RPS3a-like* mRNA.

### PSTVd infection induces cleavage of putative target mRNAs

**Figure 5:**
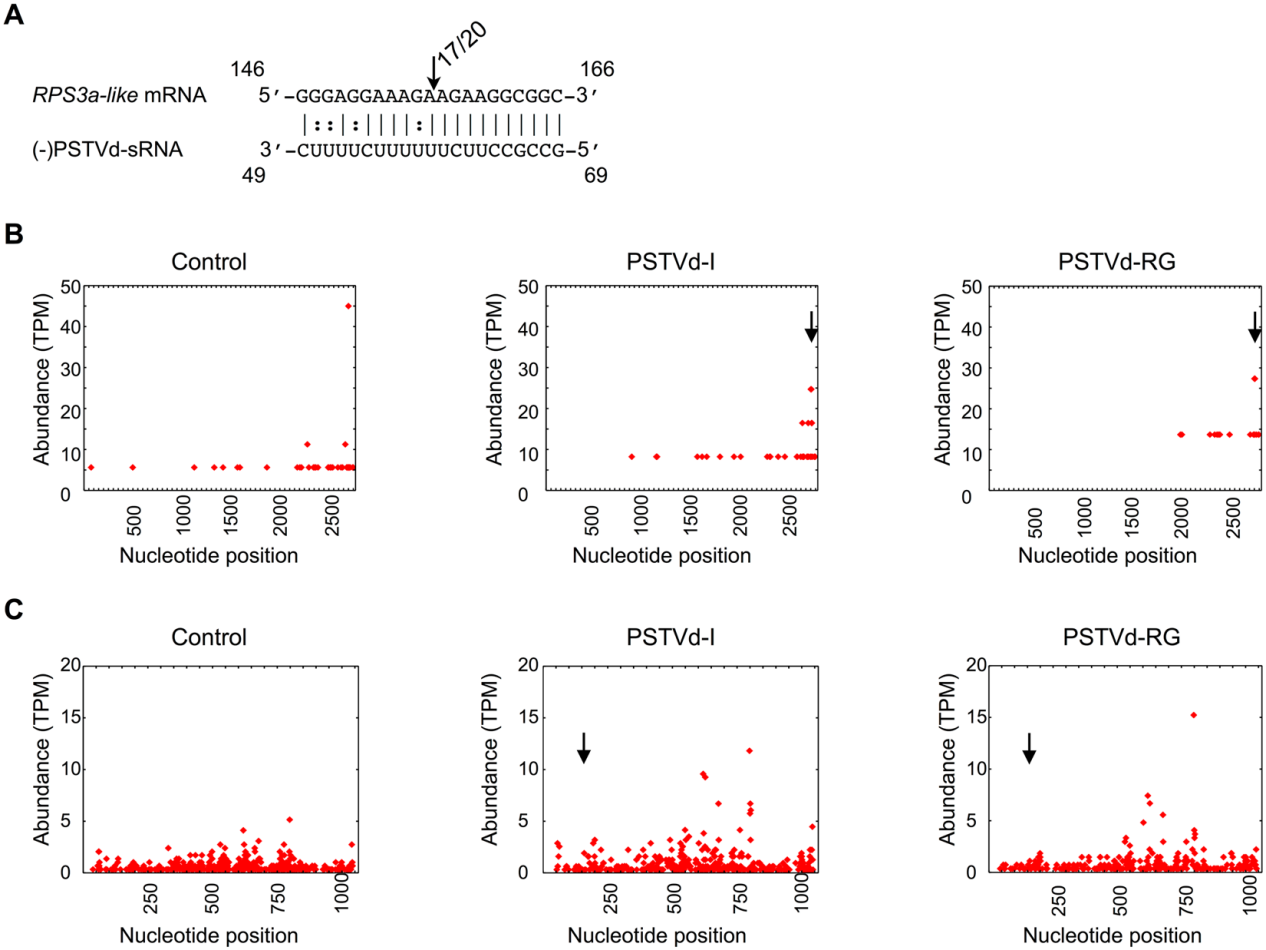
Analysis of RISC-Mediated Cleavage of the *CLC-b-like* and *RPS3a-like* mRNA by RLM RACE. (**A**) *RPS3a-like* mRNA/vd-sRNA duplexes predicted to be formed by the sRNA derived from the PSTVd variants. The arrows indicate the 5′ termini of *RPS3a-like* mRNA fragments isolated from the PSTVd-infected plants, as identified by 5′ RLM-RACE products, with the frequency of clones shown. (i.e. 17/20, indicates that 17 cleavage products were found out of 20 analyzed clones). Sequences are shown in the complementary polarity. The PARE reads were profiled against (**B**) the *chloride channel protein CLC-b-like* and (**C**) the *RPS3a-like* mRNAs. The vertical arrow denotes the predicted cleavage site. All of the reads were normalized to Transcripts Per Million (TPM). Note that different scales were used.

Conventionally, 5′ RNA ligase mediated rapid amplification of cDNA ends (5′ RLM RACE) is the widely accepted technique to confirm the RISC-mediated cleavage of a target RNA^30^. The same technique has been applied in order to validate the cleavage of predicted target mRNAs in viroid infected plants^10–12^. The RISC-mediated cleavage of predicted target sequence of *RPS3a-like* mRNA was verified by performing a 5′ RLM RACE experiment. However, vd-sRNA which was predicted to bind 3′ UTR of *chloride channel protein CLC-b-like mRNA*, leaves little space to design primers for 5′ RLM RACE. Hence, the RISC-mediated cleavage of predicted target sequence of *chloride channel protein CLC-b-like* mRNA was not evaluated by 5′ RLM RACE experiment. The cDNAs were produced in 5′ RLM RACE by ligating an RNA linker to the free 5′-phosphate end of an uncapped mRNA followed by reverse transcription using oligo(dT) primer. The ligation products were subsequently amplified by nested PCR, cloned and sequenced. To verify RISC-mediated cleavage at the predicted site, RLM-RACE experiment was performed on the 7-dpi RNA preparations using a gene-specific nested PCR. The resulting amplicons of expected size were cloned and sequenced. The cDNA clones obtained from the *RPS3a-like* transcripts at 7-dpi were aligned to detect the 5′ termini. Of the twenty obtained clones seventeen had a single cleavage site at 5′ termini that was identical to the predicted cleavage site (Fig. 5A). No PCR amplification was obtained when similar experiments were performed with RNA preparations obtained from mock-inoculated plants, indicating specific cleavage of the *RPS3a*-like mRNA by the PSTVd-sRNA. The data presented here validate the cleavage of the predicted target *RPS3a*-like mRNA sequence in PSTVd-infected plants.

In order to verify whether viroid infection induces widespread degradation of putative target mRNA, the same RNA samples (21-dpi) that were used for the analysis of the viroid accumulation levels were used for a Parallel analysis of RNA ends (PARE) experiment, as described previously with modifications^31^. The PARE data obtained were then aligned against the whole lengths of both the *chloride channel protein CLC-b-like* and the *RPS3a-like* mRNAs, and the sequences that showed homologies were retrieved. Plotting of these retrieved sequences against the respective mRNAs after normalization revealed widespread cleavage throughout the lengths of the mRNAs (Fig. 5B,C). Visual analysis of the *Chloride channel CLC-b-like* mRNA plots obtained for PSTVd-I and PSTVd-RG1 infected plants exhibited a similar pattern albeit with more cleavages located at the 3′ end. It is worth noting that profiling of the PARE data on PSTVd-RG1 showed less cleavage as compared to that seen in control plants. This can be attributed to the fact that the control plants had more than double the amount of PARE sequences as compared to those in the PSTVd-RG1 inoculated plants. On the other hand, the *RPS3a-like* mRNA exhibited a high degree of cleavage throughout the length of the mRNA, irrespective of sample, but the degradation level was higher in viroid infected plants than in the control plants. These retrieved PARE sequences were BLAST analyzed in order to see if there was any sequence similarity with the (+) and (−) strands of PSTVd. If there was any sequence homology, the mRNA wide cleavage might have occurred due to vd-sRNA:mRNA interaction. None of the sequences showed any sequence similarities with PSTVd other than the predicted ones. Taken together, data presented here clearly shows that viroid infection indirectly induces the degradation of endogenous mRNA.

## Discussion

Viroids are both potential inducers and targets of RNA silencing due to their highly base-paired circular ssRNA genome, and to the fact that they replicate through a rolling circle mechanism that involves dsRNA replicative intermediates^32^. Due to this RNA-RNA base pairing, viroids produce both (+) and (−) strand specific sRNAs. Previously, we have shown that sRNA derived from virulence modulating region (VMR) of PSTVd induces can down-regulate callose synthase genes *CalS11-like* and *CalS12-like* by RNA silencing. The products of *CalS11-like* and *CalS12-like* are essential for the formation of callose during pathogen infection, hence plays important role in plant’s defense by preventing systemic spreading of the pathogen. By performing mutatgenic studies on PSTVd, we verified the role of VMR derived vd-sRNA on the viroid accumulation. The polymorphism in the vd-sRNA binding region of *CalS11-like* mRNA greatly affected viroid accumulation in different tomato cultivars^12^. In the present study, to understand the mechanism behind the observed higher level of down-regulation of endogenous mRNAs in the presence of fewer amounts of recovered target binding vd-sRNAs, the two least expressed vd-sRNA of both the PSTVd (+) and (−) strands were selected for an *in silico* analysis that searched for possible RNA:RNA interactions with the tomato transcriptome^19^. This search revealed the possibility of vd-sRNA:mRNA duplex formation with both the 3′ UTR of the *chloride channel protein CLC-b-like* mRNA and the coding region of the *RPS3a* mRNA (Fig. 1). Both putative target gene sequences used in the studies are from genome sequenced tomato cultivar Heinz 1706. Though tomato cultivar Rutgers is a conventional experimental system for studying tomato-PSTVd interactions, its genome has not been sequenced. Hence, sequence of potential target sites in tomato cultivar Rutgers was confirmed by sequence analysis. As product of the *chloride channel protein CLC-b-like* gene is involved in important physiological functions such as cell volume regulation, signal transduction and *trans-epithelial* transport; while that of the *RPS3a-like* gene forms the structural component of the 40S ribosomal RNA, making it an extremely interesting subject. The effect of these mRNAs on host’s phenotype was evaluated by VIGS mediated knock-down assay (Fig. 2). The RNA silenced plants revealed stunting and leaf curling, suggesting their possible role in the PSTVd induced pathogenesis.

In order to evaluate the effect of the viroid’s titer on the predicted target mRNAs in tomato plants, two variants of PSTVd were selected based on their known abilities to cause severe symptoms in tomato plants^9, 33^. A time course experiment of the PSTVd titer revealed that PSTVd-RG1 accumulated at a faster pace than did PSTVd-I (Fig. 3B). In the infected plants, PSTVd-RG1 down-regulated both the *chloride channel protein CLC-b-like* and *RPS3a-like* mRNAs, while PSTVd-I infected plants revealed the down-regulation of only the *RPS3a-like* mRNA, at 14-dpi (Fig. 3C and D). This may be due to the lower accumulation level of PSTVd-I, as compared to that of PSTVd-RG1. Analyses of the large-scale sequence data obtained for the 21 to 24-nt long vd-sRNAs produced by both PSTVd-I and PSTVd-RG1 in inoculated plants showed accumulation of vd-sRNA. This large-scale data also confirmed the production of specific vd-sRNAs which were predicted to target both the *chloride channel protein CLC-b-like* and the *RPS3a-like* mRNAs in tomato plants. The recovery of a fewer number of total vd-sRNA in the present study can be attributed to the fact that an Illumina Mi-Seq platform was used for the large-scale sequencing which resulted in fewer total reads as compared to previous studies where the Illumina Hi-Seq platform was used^8, 9, 34^.

Over-expression of vd-sRNA by amiRNA assays helps to verify the direct interaction between vd-sRNA and its putative target sequence^12, 29, 35^. The amiRNA experiment performed for the vd-sRNA:*chloride channel protein CLC-b-like* and vd-sRNA:*RPS3a-like* mRNA target sequences resulted in the repression of GFP production demonstrating the direct interaction between the vd-sRNA and the putative target sequences (Fig. 4). Often, the predicted RNA:RNA duplex formation between the miRNA and the target does not lead to the cleavage of the target mRNA, but instead results in translational repression^36^. Hence, a RNA gel blot analysis was performed and it confirmed the suppression of the mRNA itself, suggesting that the cleavage of target sequence did indeed occur. In addition, comparison of the relative intensities observed for GFP expression with those of the RNA gel blot assay revealed the higher level of translational suppression than there is decrease in the RNA level. The absence of successful nested PCR reactions using RLM-RACE on RNA preparations from mock-inoculated plants helped to confirm the cleavage of the *RPS3a-like* mRNAs that are associated with PSTVd infection. Sequence analysis of the cDNA clones of *RPS3a-like* transcripts obtained from PSTVd inoculated plants confirmed the RISC-mediated cleavage sites in the *RPS3a-like* mRNA (Fig. 5A). In aggregate the two experiments validate the direct interaction between the vd-sRNA and the predicted target sequence.

5′ RLM RACE is a widely accepted technique used in order to confirm the RISC-mediated cleavage of a target mRNA^30^. Recently, 5′ RLM RACE combined with high-throughput sequencing techniques, such as the PARE, the degradome approach and the genome-wide mapping of uncapped and cleaved transcripts (GMUCT), has gained importance as these techniques permit the evaluation of a the large number of samples in a single experiment and have diverse applications for the study of the RNA degradome^6, 37, 38^. All of these methods comprise a modified 5′ rapid amplification of cDNA ends (5′ RACE) followed by deep sequencing and bioinformatics analysis in order to determine the 3′ cleaved ends of the mRNAs. As these methods use global sampling, they allow comparison of the relative abundances of the mRNAs cleaved by vd-sRNA interaction, as well as of the non-specific degradation of mRNA caused by altered state of the diseased plant. In order to verify the vd-sRNA independent degradation of endogenous mRNAs, the RNA samples extracted at 21-dpi were subjected for PARE. Analysis of PARE data revealed very few cleavages at the predicted target site of *RPS3a-like mRNA* in comparison to the data obtained by 5′ RLM RACE. This inconsistency observed between the two different experiments might be due to: (i) samples from different time of infection were used (7-dpi samples were used for 5′ RLM RACE and 21-dpi samples were used for PARE); and, (ii) viroid infection induces non-specific degradation of host mRNA in the later stages of the infection. Plotting of the PARE data against the respective target mRNA revealed the presence of cleavage sites throughout the mRNA, indicating the possibility of the induction of the degradation of host mRNA by viroid infection (Fig. 5B,C). Instances of indirect degradation of SANT/HTH-Myb (*Sl*Myb) mRNA in viroid infected plants have been demonstrated previously^39^. More recently, transcriptome analyses of PSTVd infected tomato plants have revealed genome-wide alterations^40^. Our data is consistent with those previous observations, further supporting the viroid infection induced vd-sRNA independent degradation of host mRNAs.

**Figure 6:**
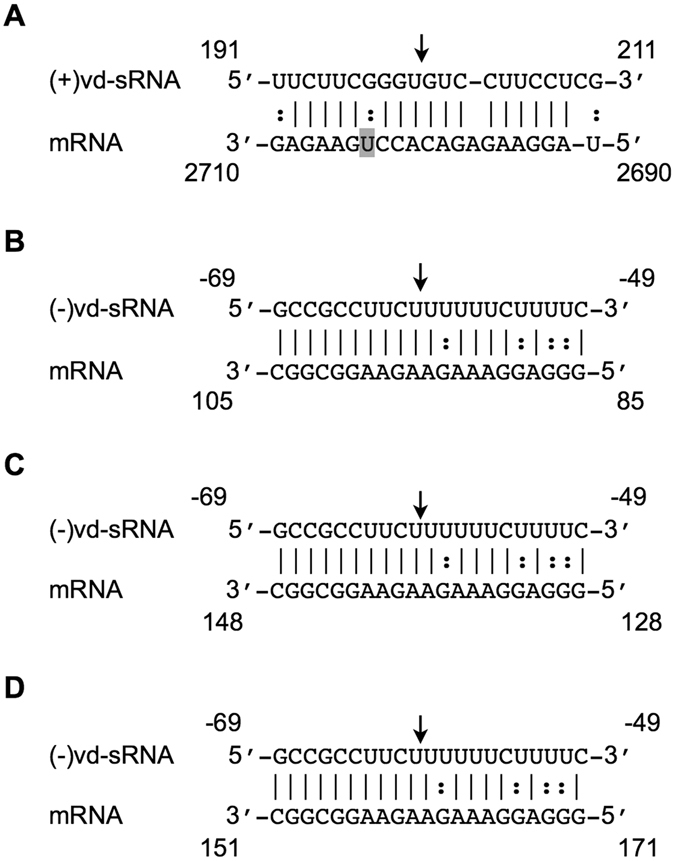
Sequence similarities between the PSTVd-sRNA binding sites of tomato and other host genomes. BLAST analyses were performed against the genomes of both potato and Nicotiana species in order to detect the presence of sequence homologies with both the *chloride channel protein CLC-b-like* and the *RPS3a-like* mRNAs of tomato as these species are the hosts of PSTVd under natural and laboratory conditions, respectively. The sequence obtained was aligned against the respective targeting vd-sRNA in order to identify any possible RISC mediated cleavage sites. The vd-sRNA targeting the *chloride channel protein CLC-b-like* mRNA in tomato was predicted to target (**A**) the potato, but not the Nicotiana species. The tomato and potato c*hloride channel protein CLC-b-like* mRNAs have one mismatch, which is shadowed. The vd-sRNA *RPS3a-like* mRNA was predicted to target both the *RPS3a-like* (**B**) and the *cyc07-like* mRNAs (**C**) in potato. The same vd-sRNA can also target the *RPS3a-like* mRNA (**D**) in *Nicotiana sylvestris*. The arrows indicate the predicted cleavage sites.

Although tomato and potato plants are natural hosts of PSTVd, tomato is the widely used experimental host plant due to its convenience. As both species belong to the same genus, high degrees of sequence homologies between their *chloride channel protein CLC-b-like* and their *RPS3a-like* genes were suspected. A BLAST search of the vd-sRNA binding sites of the tomato *chloride channel protein CLC-b-like* mRNA against the potato genome revealed 95% sequence identity (GenBank Acc. No. XM_006357128). The sequence alignment of the vd-sRNA binding site of potato against positions 191 to 211 of the vd-sRNA revealed the presence of a mismatch located 9 nt from the 5′ end of the vd-sRNA (Fig. 6A). This mismatch may result in inefficient RNA silencing in potato plants as compared to that seen in tomato plants. In contrast, a BLAST analysis of the vd-sRNA binding site of the *RPS3a-like* mRNA of the tomato against the potato genome revealed a 100% sequence identity (GenBank Acc. No. NM_001287984). As a consequence, it forms an RNA:RNA duplex similar to that of the *RPS3a-like* mRNA of tomato (Fig. 6B). Surprisingly, the same region of the *RPS3a-like* mRNA showed 100% sequence similarity with the *cyc07-like* protein mRNA of potato, and it therefore exhibits the same vd-sRNA:target mRNA duplex pattern as the *RPS3a-like* mRNA (GenBank Acc. No. NM_001288194; Fig. 6C). The *cyc07-like* protein gene encodes the structural constituent of the ribosome and is thus involved in the translational process (http://www.uniprot.org/uniprot/Q2XPX9). A BLAST analysis of the vd-sRNA binding site of the *cyc07-like* protein mRNA against the tomato genome did not reveal any homology. Additionally, the search for possible 5′ RACE ends in the tomato PARE library did not reveal any sequence identity, indicating the possibility of sequence diversity in the *cyc07-like* protein gene among tomato and potato cultivars. *Nicotiana* species are the other widely used laboratory plant for viroid research. A BLAST analysis of the vd-sRNA binding site of the *chloride channel protein CLC-b-like* mRNA against the *Nicotiana* genome did not reveal the presence of any sequence similarities, whereas the vd-sRNA binding site of the *RPS3a-like* mRNA of tomato possessed a 100% sequence similarity with *Nicotiana sylvestris* (*N. sylvestris*), suggesting a possible vd-sRNA:target RNA duplex formation in this plant (GenBank Acc. No. XM_009777066; Fig. 6D). These data also suggest the possibility of the targeting of the same gene in different susceptible plants by the same vd-sRNA.

The data presented here clearly demonstrates how the vd-sRNAs derived from both the (+) and the (−) strands of PSTVd have a potential to impact transcriptome, even though these vd-sRNAs are present in low numbers in infected plants. Additionally, the PARE data presented using different PSTVd variants, showed that vd-sRNAs are not the sole culprit in viroid pathogenicity. These findings increase our comprehension of the molecular basis of viroid etiology, a concept which to date has not been understood to its depth.

## Materials and Methods

### PSTVd cDNA construction and transcription

The head-to-tail dimeric constructs of both PSTVd-I (GenBank Acc. No. AY937179) and PSTVd-RG1 (GenBank Acc. No. U23058) were created in the pBluseScript KS (+) vector (Stratagene) using monomeric PSTVd-I, PSTVd-RG1 and PSTVd-M obtained from gBlock Gene Fragments (Integrated DNA Technologies, Inc) as described previously^3, 12^. Recombinant plasmids were linearized with the appropriate restriction enzymes, and were then transcribed using T3 RNA polymerase. The resulting products were analyzed by electrophoresis in 1.0% agarose gels containing 1X TAE buffer (40 mM Tris, 20 mM acetic acid (pH 8.0) and 1 mM EDTA).

### Plant material and growing conditions

The tomato plants (*S. lycopersicum* cv Rutgers; Livingston Seed Co, Ohio, USA) used for the viroid inoculation assays were grown in a growth chamber at 25 °C with 16 h of light and 8 h of darkness. After the development of the primary leaves, the plants were inoculated with the 1 µg of RNA transcripts of respective viroid variants. The tomato plants used for the VIGS assay were grown at 22 °C with 16 h of light and 8 h of darkness. After the development of the primary leaves, the cotyledons were agroinfiltrated with either pTRV1 and pTRV2, or with their derivatives as described previously^28^. The *N. benthamiana* plants used for the amiRNA experiments were grown at 23 °C with 16 h of light and 8 h of darkness and the agroinforltration was performed as described previously^12^.

### Construction of the plasmids for the VIGS

The pTRV1 and pTRV2 vectors for the VIGS assay were procured from Arabidopsis Biological Resource Centre (Ohio, USA). In order to produce the pTRV2 derivative targeting either the *PDS* mRNA, the *chloride channel protein CLC-b-like* or the *RPS3a-like* mRNAs, approximately 400-bp of the respective genes were amplified from tomato cv. Rutgers using gene specific primers after extracting total nucleic acids from tomato plants according to Adkar-Purushothama *et al*.^41^. The PCR products were ligated into the pGEM-T vector (Promega, MA), and resulting plasmids were then digested with the restriction endonucleases *Xba*I and *Bam*HI. The digestion products were ligated into the same restriction sites of the binary vector pTRV2. The resulting binary vectors were then transformed into the *Agrobacterium tumefaciens* strain GV3101, and were used for agro-infiltration as previously described^28^. Details of oligonucleotides used are available upon request.

### RNA extraction and RT-qPCR

Total RNA from infected leaf samples was extracted using the *mir*Vana^TM^ miRNA isolation kit (Ambion, Austin, TX, USA) and complementary DNA (cDNA) was synthesized as described previously^12^. For the evaluation of both the PSTVd titer and the gene expression levels, 10 ng of this cDNA was used in the qPCR experiments along with appropriate primer combinations. mRNAs are amplified using gene specific primers. Ubiquitin-conjugating enzyme (UBC), Transducing/WD40 repeat family protein and the ARF*-like* GTPase family protein (ASAR1) were used as house-keeping genes to normalize of the RT-qPCR results^42^. Every qPCR run included no-template primer-pair control samples, and these were consistently negative. All the oligonucleotides used are available upon request. The relative expression levels were calculated using the qBASE framework^43^. All of the RT-qPCR analyses were performed commercially at the Laboratoire de Génomique Fonctionnelle de l’Université de Sherbrooke (http://palace.lgfus.ca).

### Construction of PSTVd specific amiRNA

The amiRNA for different vd-sRNA sequences were synthesized on an osa-MIR528 backbone using appropriate oligonucleotide pairs in the PCR reaction and were ligated to the binary vector pBIN61 as described previously^29^. The target construct was prepared by ligating 21-nt long predicted target sequences to the 3′ UTR of the GFP gene in the pBIN61 vector. The resulting binary vectors were then transformed into the *Agrobacterium tumefaciens* strain C58C1 carrying the virulence plasmid pCH32, and were used for agro-infiltration as previously described^12^.

### Gel blot analysis

The riboprobes for the detection of both the GFP and the 7SL mRNAs were prepared using the GFP and the 7SL genes previously cloned in the pBlueScript KS (+) vector. The riboprobes were prepared by linearizing the pBlueScript KS (+) vectors with *Kpn*I restriction endonuclease followed by transcription using the T7 MAXIscript kit (Ambion) according to the manufacturer’s protocol. In order to detect the *chloride channel protein CLC-b-like*, the *RPS3a*-*like* and the *5S rRNA*, mRNAs, 5′ ends of the DNA oligo nucleotides were radiolabeled with [γ-^32^P]ATP in the presence of T4 polynucleotide kinase (PNK).

For the GFP-mRNA expression analysis, a northern blot was performed using radiolabelled GFP probes. Briefly, two leaf discs were homogenized in TRIzol® buffer and the RNA was extracted as per the manufacturer’s instructions (Invitrogen, Carlsbad, CA, USA). A total of 250 ng of the RNA sample was dissolved in 5 µl of a loading buffer containing 10% (v/v) glycerol and 0.01% each of bromo-phenol blue and xylene cyanol FF. The samples were heat-denatured at 65 °C for 15 min in the presence of 3 vol of sample buffer (50% formamide, 2.2 M formaldehyde [37%]), and were then were separated by electrophoresis in 1.0% agarose-formamide gels containing 1x MOPS buffer. The RNAs were then transferred to a Hybond-XL nylon membrane (Amersham, GE Healthcare Life Sciences) and hybridized with radiolabelled riboprobe at 55 °C for 16 h in order to detect the GFP. The membranes were washed twice for 20 min at 62 °C with washing buffer (1x SSC, 0.1x SDS), and the hybridization signals were then visualized using a Typhoon imaging system (GE Healthcare Life Sciences).

For the RNA gel blot hybridizations of the VIGSed plants, 2.0 μg of the total RNA extracted from 18-dpi plants were transferred to a Hybond-XL nylon membrane as described above. The transferred RNAs were hybridized with radiolabeled probes in order to detect the *chloride channel protein CLC-b*-*like*, the *RPS3a*-*like* and the *5S rRNA* mRNAs. The *chloride channel protein CLC-b*-*like* and the *RPS3a*-*like* probes were hybridized overnight at 45 °C, while the *5S* rRNA probe was hybridized at 55 °C. The membranes were washed twice with washing buffer, and the RNA gel blot hybridization signals were visualized using a Typhoon imaging system.

### Immunoblot Analysis

For GFP expression analysis, total protein was extracted from two leaf discs using 1.5X of Laemmli sample buffer as described previously^12^. Anti-GFP-HRP antibodies were used to detect GFP according to the manufacturer’s instructions (Santa Cruz Biotechnology). Anti-PEPC rabbit antibodies (Rockland Immunochemicals) were used to detect PEPC, followed by a subsequent incubation with donkey anti-IgG rabbit-HRP polyclonal antibodies (BioLegend). The proteins were subsequently visualized using ECL substrate (Bio-Rad) as per the manufacturer’s instructions. The amount of GFP produced was quantified using ImageJ software (http://imagej.nih.gov/ij). The data obtained for the housekeeping gene (PEPC) was used for the normalization.

### Small RNA library preparation and data analyses

For the high-throughput sequencing of the small RNAs (sRNAs), the small RNA of 15 to 50-nt were column purified from the total RNA extracted from 21-dpi plants, and were then subjected to sRNA library preparation using the NEBNext Multiplex Small RNA Library Prep Set for Illumina according to the manufacturer’s instructions (New England Biolabs). The products obtained were then processed simultaneously in the Illumina Mi-Seq platform using a multiplex strategy. The adapters were trimmed^44^, and only 21 to 24-nt long vd-sRNAs were filtered using a Python program^45^. The filtered 21 to 24-nt vd-sRNAs were plotted against both the (+) and the (−) strands of the respective PSTVd variants after normalizing to the Reads Per Million (RPM) scale using a standard pattern-matching algorithm developed in the Python programming language^46^.

### RNA ligase-mediated rapid amplification of cDNA ends (RLM RACE)

In order to detect the cleavage sites of the *RPS3a* mRNA, a 5′ RLM RACE was performed by ligating an RNA adaptor to the 5′-phosphate of the total RNA extracted as described previously (12). The resulting product was reverse transcribed using Oligo (dT) primer. The products were subjected to nested PCR using *RPS3a-like* gene specific primers. The nested PCR products were separated, eluted, cloned, sequenced and analyzed for the 5′ termini as described before.

### Parallel analysis of RNA end libraries and bioinformatic analyses

For the PARE analysis, the protocol designed for the Illumina Hi-Seq platforms was modified for the Illumina Mi-Seq platform^31^. Briefly, 120 µg of total RNA obtained from 21-dpi plants were used to prepare mRNA using the GenElute mRNA Mini Prep kit according to the manufacturer’s instructions (Sigma-Aldrich). A 5′-PARE RNA adapter was ligated to the 5′-uncapped ends of the purified mRNA by incubation at 37 °C for 1 h in the presence of T4 RNA *ligase* I (New England Biolabs, Inc, MA, USA). The resulting RNA products were reverse-transcribed to cDNA using the Target RT-primer in the presence of SuperScript^®^ III *Reverse transcriptase* according to the manufacturer’s instructions (Invitrogen, Carlsbad, CA, USA). This was followed by 7 cycles of PCR amplification using both the 5′- and 3′-cDNA PCR primers. The amplicons were then subjected to *Mme* I restriction endonuclease digestion for 1 h according to the manufacturer’s instructions (New England Biolabs Inc, MA, USA). The digestion products were separated on 5% polyacrylamide gels, and the gel slices containing a band of approximately 70-bp were excised and the DNA was eluted overnight using an elution buffer (10 mM magnesium acetate tetrahydrate, 0.5 M ammonium acetate, 1 mM EDTA [pH 8.0], 0.1% [w/v] SDS). The eluted products were subjected to 15 cycles of PCR amplification with the Illumina universal primer using the multiplex strategy. The obtained products were purified and sequenced in the Illumina Mi-Seq platform at the Laboratoire de Génomique Fonctionnelle de l’Université de Sherbrooke (http://palace.lgfus.ca). The adapter sequences were trimmed, and only 20-nt long sequences were filtered as before. The filtered sequences were mapped against mRNA sequences using a standard pattern-matching algorithm developed in Python^46^. The data was normalized to TPM and mapped on the respective mRNA sequences as described previously^47^.

### Accession numbers

The deep sequence data used in this study is deposited in GEO with the Accession No. GSE70256 (sRNA derived from control plant); Accession No. GSE70096 (sRNA derived from PSTVd-I inoculated plant); GSE70166 (sRNA derived from PSTVd-RG1 inoculated plant); GSE88707 (PARE data obtained from mock inoculated plants); GSE70037 (PARE data obtained from PSTVd-I inoculated plants) and GSE70062 (PARE data obtained from PSTVd-RG1 inoculated plants).

## Electronic supplementary material

Supplemental data
