# Supplementary material for: Potato spindle tuber viroid infection triggers degradation of chloride channel protein CLC-b-like and Ribosomal protein S3a-like mRNAs in tomato plants
Source: Sci Rep. 2017 Aug 21;7:8341. doi: 10.1038/s41598-017-08823-z (PMC5566334; doi:10.1038/s41598-017-08823-z)
Supplement: Supplementary file 1 — Supplemental data [file 41598_2017_8823_MOESM1_ESM.pdf]

Supplemental information

***Potato spindle tuber viroid* infection triggers *degradation of chloride channel protein CLC-b-like* and *Ribosomal protein S3a-like* mRNAs in tomato plants**

Charith Raj Adkar-Purushothama[1*], Pavithran Sridharan Iyer[2] and Jean-Pierre Perreault[1*]

[1]RNA Group/Groupe ARN, Département de Biochimie, Faculté de médecine des sciences de la santé, Pavillon de Recherche Appliquée au Cancer, Université de Sherbrooke, 3201 rue JeanMignault, Sherbrooke, Québec, J1E 4K8, Canada

[2]Département de Physique, Université de Sherbrooke, 2500 Boulevard de l'Université Sherbrooke, Québec, J1K 2R1, Canada.

*Corresponding authors

E-mails: charith.adkar@USherbrooke.ca (CRA)

Jean-Pierre.Perreault@USherbrooke.ca (JPP)

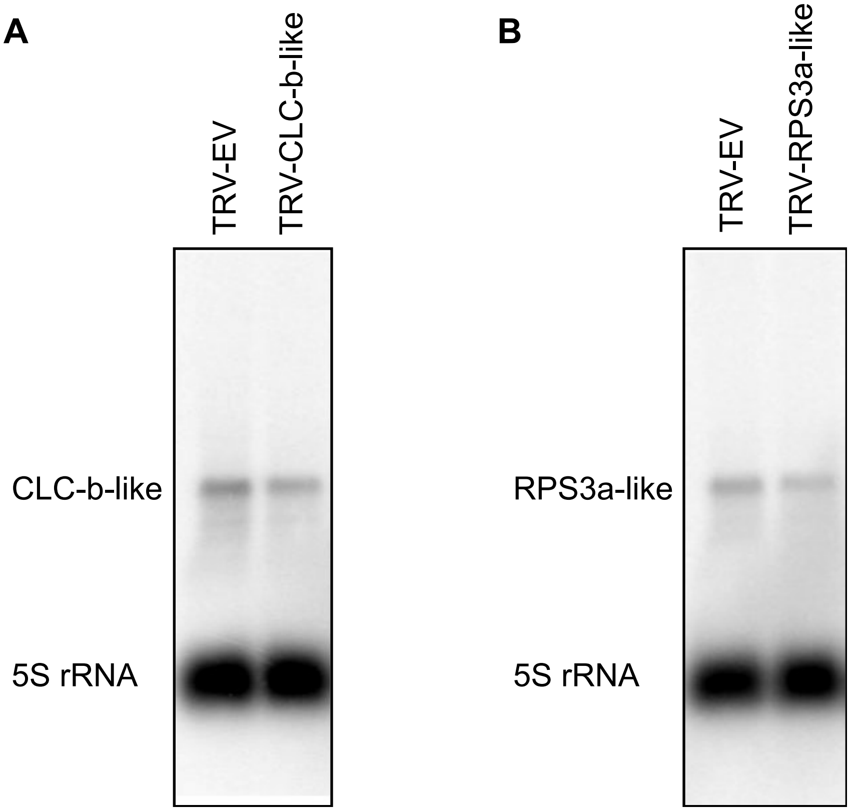

Fig S1: VIGS mediated knock-down of chloride channel protein CLC-b-like mRNA and the 40S ribosomal protein S3a-like mRNA.

The tomato plants were subjected to a knock-down assay by a VIGS technique using the TRV1 vector in combination with either the TRV2 empty vector, or with its derivatives. After 18-days post infiltration, total RNA extracted from the agroinfitrated plants were analyzed by gel blot assay for the knock-down/suppression of (**A**) *Chloride channel CLC-b*-like and, (**B**) *RPS3a*-like mRNAs using gene specific radiolabeled probes. The *5S* rRNA was used as a loading control.

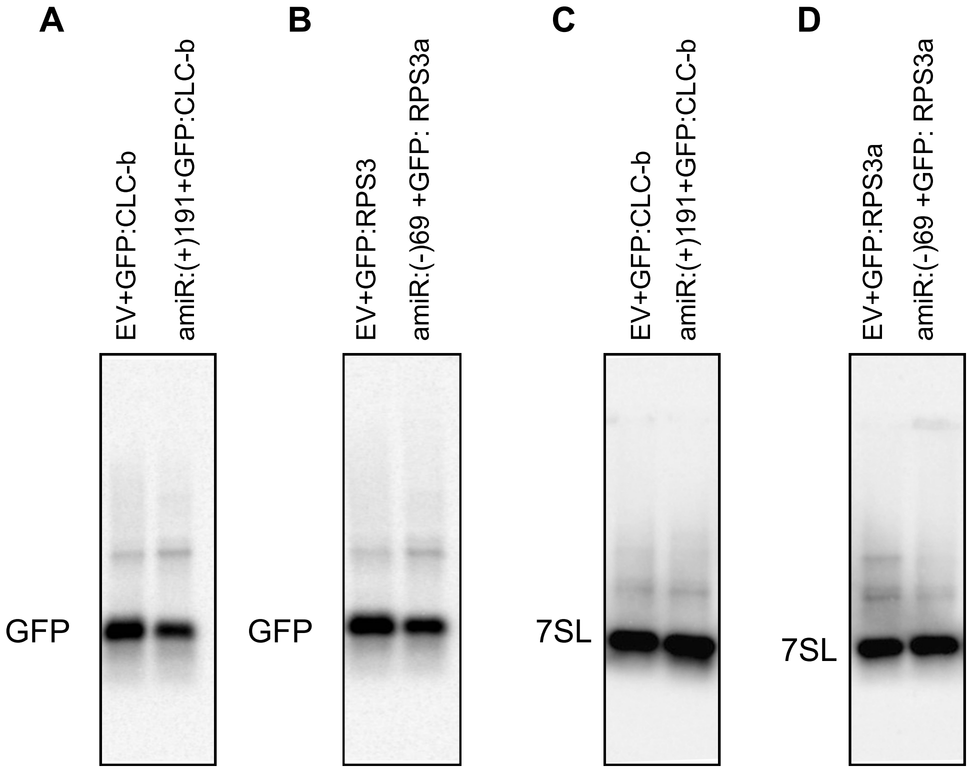

Figure S2: Validation of the reduction in the amount of GFP-mRNA in the various vd-sRNA:amiRNA plus GFP:target combinations.

N. benthamiana leaves were agro-infiltrated with empty pBIN61 vector (EV) plus GFP:CLCb; amiR:(+)191 plus GFP:CLCb; EV plus GFP:RPS3a; and, amiR:(-)69 plus GFP:RPS3a. At 3-dpi, total RNA extracts were subjected to RNA gel blot analyses with either (**A**) and (**B**) GFP or (**C**) and (**D**)*7SL* radiolabeled probes.

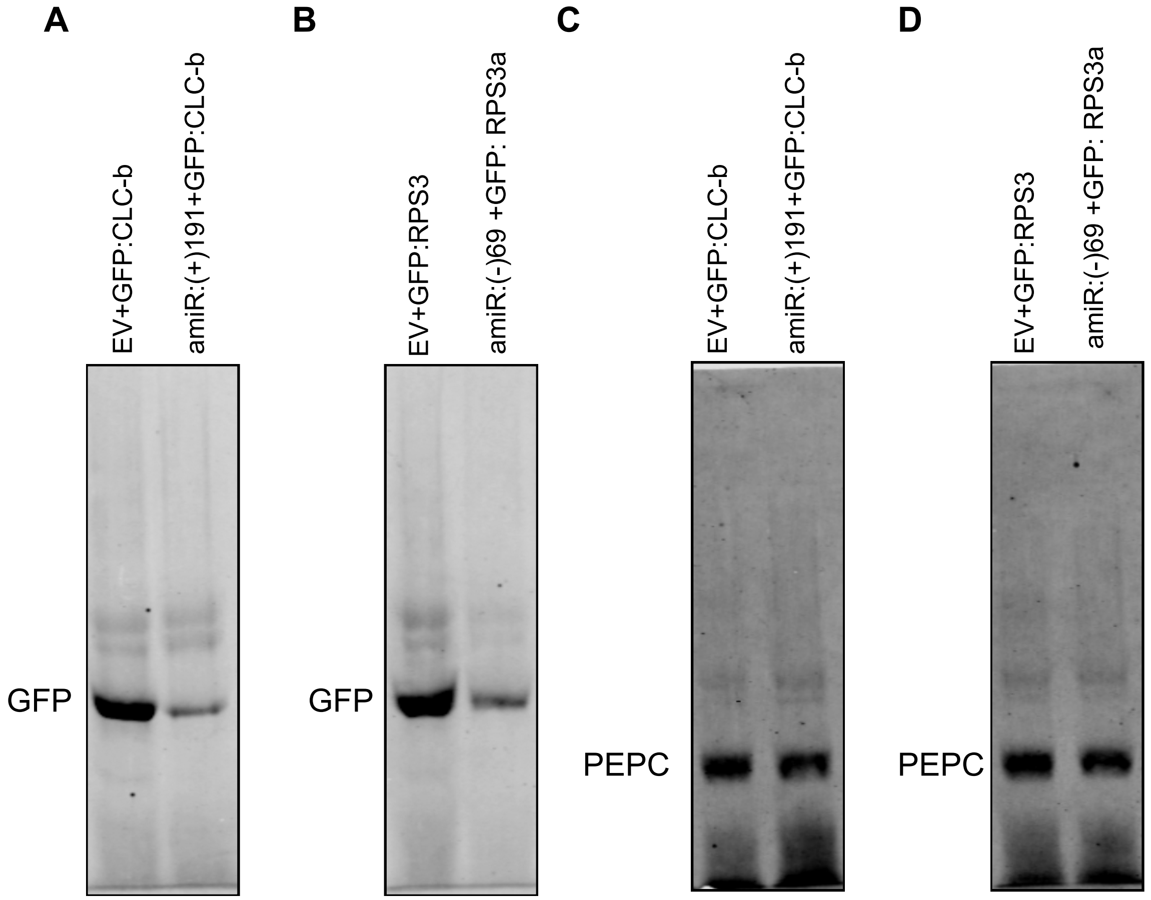

Figure S3: Validation of the translational repression of GFP-mRNA in the various vd-sRNA:amiRNA plus GFP:target combinations.

N. benthamiana leaves were agro-infiltrated with empty pBIN61 vector (EV) plus GFP:CLCb; amiR:(+)191 plus GFP:CLCb; EV plus GFP:RPS3a; and, amiR:(-)69 plus GFP:RPS3a. At 3 dpi, total protein extracts were subjected to immunoblotting with anti-GFP (A and B) and anti-PEPC (C and D) antibodies.
